# Short- and Long-Term Outcomes after Pancreatectomy for Pancreatic Cancer in Patients with Prior Esophagectomy for Esophageal Cancer

**DOI:** 10.1245/s10434-025-18491-3

**Published:** 2025-10-14

**Authors:** Jun Ishida, Yoshihide Nanno, Hirochika Toyama, Takuya Mizumoto, Dongha Lee, Takeshi Urade, Kenji Fukushima, Shohei Komatsu, Sadaki Asari, Hiroaki Yanagimoto, Masahiro Kido, Takumi Fukumoto

**Affiliations:** 1 Division of Hepato-Biliary-Pancreatic Surgery, Department of Surgery, Kobe University Graduate School of Medicine, Kobe, Japan; 2 Department of Surgery, Osaka Saiseikai Nakatsu Hospital, Osaka, Japan

**Keywords:** Pancreatic ductal adenocarcinoma, Pancreaticoduodenectomy, Distal pancreatectomy, Esophagectomy

## Abstract

**Background:**

Resection of pancreatic ductal adenocarcinoma (PDAC) after prior esophagectomy for esophageal cancer is technically demanding and its oncologic value is unclear. We evaluated the peri-operative safety and long-term survival after pancreatectomy for PDAC in this setting.

**Methods:**

All patients undergoing pancreatectomy for PDAC at Kobe University Hospital (2017–2024) were reviewed. Outcomes in patients with previous esophagectomy for esophageal cancer were compared with those in patients undergoing contemporaneous resectable PDAC without such history. Primary endpoints were postoperative morbidity/mortality and overall survival.

**Results:**

Of 415 pancreatectomies, nine (2.2%) had a history of esophagectomy for esophageal cancer (pancreaticoduodenectomy five; distal pancreatectomy four). Preservation of the gastrointestinal-conduit vessels was achieved in seven patients; two required right gastroepiploic artery and vein reconstruction. Major morbidity occurred in one patient (11.1%); there was no 90-day mortality and no conduit-related complication. However, only three patients (33.3%) received adjuvant chemotherapy. Median overall survival was significantly shorter in post-esophagectomy patients (n = 9) than in controls (n = 249) (8.4 vs. 41.7 months, *p* = 0.001). Survival after pancreaticoduodenectomy was especially poor (7.4 vs. 47.2 months, *p* < 0.001), whereas outcomes after distal pancreatectomy did not differ (16.4 vs. 38.9 months, *p* = 0.781).

**Conclusions:**

Pancreatectomy after esophagectomy can be performed safely, even with minimally invasive techniques, yet long-term survival remains dismal, particularly after pancreaticoduodenectomy. Careful patient selection with specific attention to the feasibility of delivering perioperative chemotherapy may be required to improve outcomes in this highly selected cohort.

**Supplementary Information:**

The online version contains supplementary material available at 10.1245/s10434-025-18491-3.

Pancreatic ductal adenocarcinoma (PDAC) remains an aggressive malignancy with a 5-year survival rate of approximately 13%.^1^ Surgical resection offers the only potential cure. Recent advances in surgical technology and perioperative chemotherapy have improved both short- and long-term outcomes after pancreatectomy.^2^ Concomitantly, an increasing number of patients present with PDAC after previous abdominal surgery, reflecting improved survival after other gastrointestinal cancers, including esophageal, gastric, and hepatobiliary malignancies.^3,4^

Esophagectomy is the standard treatment for esophageal cancer unsuitable for endoscopic submucosal dissection.^5^ Pancreatectomy after esophagectomy is technically challenging because the vascular supply to the gastrointestinal conduit must be preserved. Gastric conduit reconstruction is the most common method following esophagectomy.^6^ In patients with PDAC of the pancreatic head after gastric conduit reconstruction, preservation of the right gastroepiploic artery is mandatory but often difficult because the tumor is frequently adjacent to the gastroduodenal artery.^7^

Although several case reports have described pancreatectomy for PDAC after esophagectomy for esophageal cancer,^7–10^ robust evidence regarding its safety and oncologic efficacy is lacking. We therefore conducted a single-center retrospective study to evaluate the short- and long-term outcomes of pancreatectomy for PDAC in patients with a history of esophagectomy for esophageal cancer.

## Methods

### Study Design

Patients who underwent pancreatectomy for PDAC between 2017 and 2024 at Kobe University Hospital were reviewed. Clinical data were extracted from a prospectively maintained database. This study investigated two primary endpoints: (1) short-term postoperative morbidity and mortality following pancreatectomy in patients with a prior esophagectomy for esophageal cancer and (2) long-term oncologic outcomes obtained by comparing these patients with counterparts who had not undergone esophagectomy. This retrospective study was approved by the institutional review board in June 2025 (approval number: B250041). Written informed consent was obtained from all patients before initiation of the study.

### Preoperative Evaluation and Treatment

All patients underwent dynamic contrast-enhanced multidetector computed tomography (MDCT), gadolinium-ethoxybenzyl-diethylenetriamine-pentaacetic acid–enhanced magnetic resonance imaging, and positron-emission tomography. Resectability (resectable, borderline-resectable, locally advanced, or metastatic) was assessed according to National Comprehensive Cancer Network guidelines by a multidisciplinary tumor board comprising pancreatic surgeons, gastroenterologists, radiologists, and medical oncologists. Tumor invasion of the gastrointestinal conduit vasculature was evaluated on MDCT. Preoperative chemotherapy or chemoradiotherapy was administered according to board recommendations.

### Operative Procedure

For tumors in the head of the pancreas, pancreaticoduodenectomy with regional lymph node resection was performed. When the vessels of the reconstructed digestive tract (e.g., gastric conduit) were not invaded by tumor, preservation of them was attempted. Resection and reconstruction of the vessels of the reconstructed digestive tract were performed when tumor invasion occurred. For tumors in the body and tail of the pancreas, distal pancreatectomy with regional lymph node resection was performed. Minimally invasive (laparoscopic or robotic) approaches were adopted when appropriate.

### Postoperative Treatment and Surveillance

Adjuvant chemotherapy with S-1 was recommended unless contraindicated by performance status or patient preference. Postoperative surveillance comprised MDCT and serum tumor-marker assessment every 3 months.

### Variables and Definitions

Demographic variables included sex, age, neoadjuvant therapy, and interval between esophagectomy and pancreatectomy. Operative variables included procedure type, minimally invasive approach, operative time, estimated blood loss, conduit-vessel preservation or reconstruction, morbidity, and 90-day mortality. Postoperative variables included pathological data of PDAC, administration of adjuvant chemotherapy, tumor recurrence, and survival time. Postoperative morbidity was assessed according to the Clavien-Dindo classification.^11^ Pathological stage was assessed according to the 8th edition of the Union for International Cancer Control staging system.

### Statistical Analysis

Continuous variables were expressed as median (range) and compared using the Mann–Whitney U-test. Categorical variables were compared using the chi-squared test. Survival was estimated using the Kaplan–Meier method and compared with the log-rank test. Statistical significance was set at *p* < 0.05. All analyses were performed using JMP version 14.0.0 (SAS Institute, Cary, NC, USA).

## Results

### Operative Outcomes

During the study periods, 415 patients underwent pancreatectomy for PDAC. Of these, nine (2.2%) had a history of esophagectomy for esophageal cancer. Table 1 shows the operative outcomes. Five patients (55.5%) underwent pancreaticoduodenectomy, and four patients (44.4%) underwent distal pancreatectomy. Three patients underwent pancreaticoduodenectomy with preservation of the vessels of the gastrointestinal conduit (gastric conduit, n = 2; jejunal conduit, n = 1). Two patients underwent pancreaticoduodenectomy with resection and reconstruction of the right epiploic vessels for preservation of gastric conduit. All four patients with distal pancreatectomy had preservation of the vessels of the gastrointestinal conduit.

**Table 1:** Operative outcomes

No.	Age	Reconstruction after ex	Procedure	Operative time (min)	Estimated blood loss (mL)	Preservation or resection of vessels of the gastrointestinal conduit	Morbidity	CD grade	90-day Mortality
1	72	Gastric conduit	OPD	525	320	Preservation of A and V	Deep SSI	2	None
2	59	Gastric conduit	OPD	638	1045	Resection and reconstruction of A and V	None	0	None
3	77	Gastric conduit	OPD	685	500	Resection and reconstruction of A and V	None	0	None
4	80	Jejunal conduit	OPD	523	750	Preservation of A and V	CRBSI	2	None
5	79	Gastric conduit	LAPD^a^	458	500	Preservation of A and V	PPH Chyle leak	3a	None
6	60	Gastric conduit	LDP	310	50	Preservation of A and V	None	0	None
7	76	Gastric conduit	LDP	345	100	Preservation of A and V	None	0	None
8	76	Gastric conduit	LDP	423	10	Preservation of A and V	None	0	None
9	73	Gastric conduit	RDP	290	10	Preservation of A and V	None	0	None

A, artery; CRBSI, catheter-related blood stream infection; Ex, esophagectomy; LAPD, laparoscopic-assisted pancreaticoduodenectomy; LDP, laparoscopic distal pancreatectomy; OPD, open pancreaticoduodenectomy; PPH, post-pancreatectomy hemorrhage; RDP, robotic distal pancreatectomy; SSI, surgical site infection; V, vein

^a^Resection was performed laparoscopically and reconstruction was performed via a mini-laparotomy

Major morbidity (Clavien–Dindo ≥3a) occurred in one patient (11.1%): a postoperative jejunal-artery hemorrhage unrelated to postoperative pancreatic fistula successfully treated by interventional radiology after laparoscopic-assisted pancreaticoduodenectomy. There was no postoperative morbidity related to the preserved gastrointestinal conduit. There was no 90-day mortality.

### Pathological Outcomes and Prognosis After Pancreatectomy

Pathologic findings and survival outcomes are shown in Table 2. The median interval time between esophagectomy and pancreatectomy was 2.8 years. Seven patients (77.8%) had stage 2B disease. R0 resection was achieved in seven patients (77.8%). Three patients (33.3%) received adjuvant chemotherapy. Tumor recurrence developed in six patients (66.7%), most commonly in the liver (55.6%). Five patients died of tumor recurrence, likely attributable to PDAC, and one patient died of heart failure.

**Table 2:** Pathological outcomes and prognosis

No	Pathology of esophageal cancer	Interval between Ex and Px (year)	Neoadjuvant chemotherapy	Pathology of PDAC	Stage of PDAC	R status of PDAC	Adjuvant chemotherapy	Recurrence	Prognosis (month)	Cause of death
1	SCC	2.4	None	Mod	2B	R1	None	Liver	Dead (7.7)	PDAC
2	SCC	1.8	None	Mod	2B	R0	None	Liver	Dead (7.1)	PDAC
3	SCC	7.5	None	Mod	2B	R0	None	Liver, LN	Dead (8.4)	PDAC
4	SCC	5.9	None	Mod	2B	R1	None	None	Dead (6.8)	Heart failure
5	SCC	16.1	None	Mod	1A	R0	None	None	Alive (5.3)	–
6	SCC	1.1	None	Por	2B	R0	S1	Liver	Dead (16.4)	PDAC
7	BSC	2.8	None	Wel	2B	R0	S1	LN	Alive (52.9)	–
8	SCC	3.1	None	Por	1B	R0	None	Liver	Dead (15.0)	PDAC
9	SCC	2.0	GS	Wel	2B	R0	S1	None	Alive (6.4)	–

BSC, basaloid-squamous carcinoma; Ex, esophagectomy; GS, gemcitabine + S1; LN, lymph node; Mod, moderately differentiated; PDAC, pancreatic ductal adenocarcinoma; Por, poorly differentiated; Px, pancreatectomy; SCC, squamous cell carcinoma; Wel, well differentiated

**Fig. 1:**
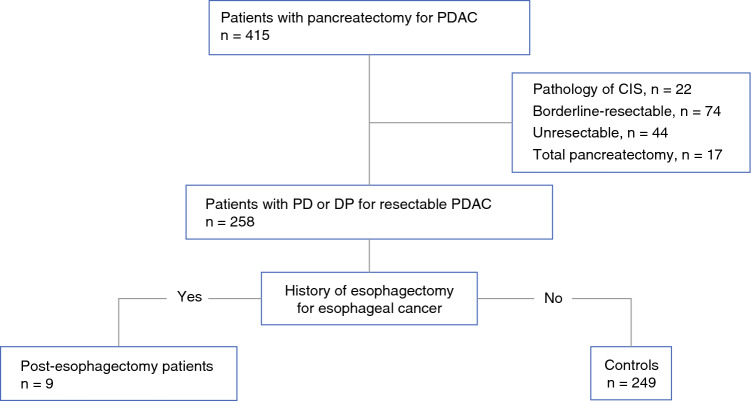
Patient flow chart for comparative analysis of long-term outcomes between patients with and without a history of esophagectomy for esophageal cancer. CIS, carcinoma in situ; DP, distal pancreatectomy; PD, pancreaticoduodenectomy; PDAC, pancreatic ductal adenocarcinoma

After excluding patients with carcinoma in situ, borderline-resectable/initially unresectable disease, and total pancreatectomy, 249 contemporaneous resectable PDAC patients without prior esophagectomy served as controls (Fig. 1). Table 3 compares the clinicopathological features of patients undergoing pancreaticoduodenectomy or distal pancreatectomy for resectable PDAC according to a history of esophagectomy for esophageal cancer. Median age was similar between the groups (76 years [range 59–80] vs. 73 years [range 39–90]; *p* = 0.634), but the post-esophagectomy patients were predominantly male (88.9% vs. 54.2%; *p* = 0.027). Pre-operative nutritional status was inferior in post-esophagectomy patients, reflected by a lower body mass index (18.5 [range 14.6–22.6] vs. 22.0 [range 12.7–31.8]; *p* = 0.003) and a lower median serum albumin concentration (3.7 g/dL [range 2.1–4.9] vs. 4.0 g/dL [range 1.9–5.0]; *p* = 0.043). Tumor-related factors—pre-operative CA19-9 (69 vs. 56 U/mL; *p* = 0.641), pathological tumor size (28 vs. 25 mm; *p* = 0.631), lymph-node involvement (77.8 vs. 55.0%; *p* = 0.162), and R0 resection rate (77.8 vs. 90.4%; *p* = 0.509)—were comparable. Administration of neoadjuvant chemotherapy showed no significant difference (11.1% vs. 28.9%; *p* = 0.204), whereas receipt of adjuvant chemotherapy was less frequent in post-esophagectomy patients (33.3% vs. 67.9%; *p* = 0.038).

**Table 3:** Clinicopathological characteristics of patients with pancreaticoduodenectomy (PD) or distal pancreatectomy (DP) for resectable pancreatic ductal adenocarcinoma (PDAC)

Variables	Post-esophagectomy patients n = 9 (% or range)	Controls n = 249 (% or range)	*p*-value
Age, year	76 (59–80)	73 (39–90)	0.634
Sex, male	8 (88.9)	135 (54.2)	**0.027**
Body mass index	18.5 (14.6–22.6)	22.0 (12.7–31.8)	**0.003**
Preoperative serum albumin, g/dL	3.7 (2.1–4.9)	4.0 (1.9–5.0)	**0.043**
Preoperative CA19-9, U/mL	69 (15–736)	56 (1–4059)	0.641
Neoadjuvant chemotherapy	1 (11.1)	72 (28.9)	0.204
Pathological tumor size, mm	28 (15–42)	25 (1–90)	0.631
Pathological lymph node involvement	7 (77.8)	137 (55.0)	0.162
R0 resection	7 (77.8)	225 (90.4)	0.509
Adjuvant chemotherapy	3 (33.3)	169 (67.9)	**0.038**

Data are presented as n (%) or n (range) unless otherwise indicated

Bold values indicate statistical significance (*p* < 0.05)

**Fig. 2:**
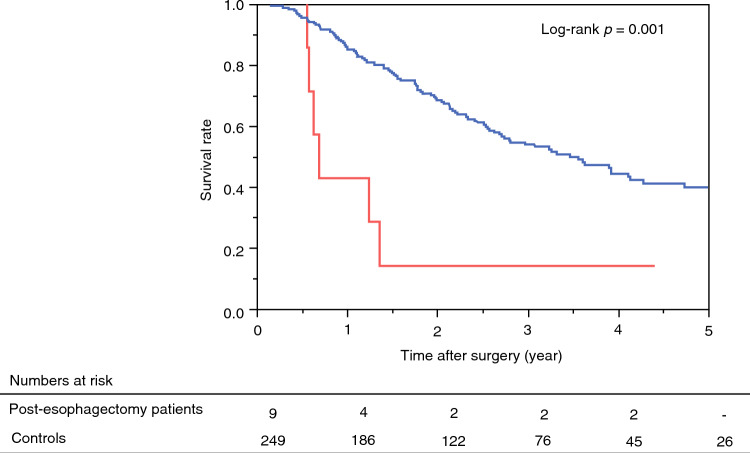
Kaplan–Meier curves of overall survival after pancreatectomy in post-esophagectomy patients (n = 9) and controls (n = 249). Red line, post-esophagectomy patients; blue line, controls

Figure 2 shows the survival curves for patients after pancreatesctomy. Median survival time (MST) after pancreatectomy in post-esophagectomy patients was 8.4 months, whereas MST after pancreatectomy in controls was 41.7 months (*p* = 0.001). Although prognosis after pancreaticoduodenectomy was significantly worse in post-esophagectomy patients than in controls (MST 7.4 vs. 47.2 months, *p* < 0.001) (Fig. S1), prognosis after distal pancreatectomy was comparable between the groups (16.4 vs. 38.9 months, *p* = 0.781) (Fig. S2).

## Discussions

This study is the first to delineate both short- and long-term outcomes of pancreatectomy for PDAC in patients who previously underwent esophagectomy for esophageal cancer. Despite the technical challenges inherent in preserving the reconstructed gastrointestinal conduit, short-term results were favorable: major morbidity occurred in only 11.1% of cases, and there were no deaths within 90 days. No complications were attributable to the preserved conduit. In contrast, long-term outcomes were poor, with a median overall survival of just 8.4 months after pancreatectomy; notably, none of the patients who underwent pancreaticoduodenectomy survived beyond 1 year post-operatively.

During pancreatectomy in patients with a prior esophagectomy, preservation of the reconstructed gastrointestinal conduit is imperative. However, in cases of PDAC in the pancreatic head with a gastric conduit, sparing the gastroepiploic vessels is often hindered by tumor invasion. We encountered two patients in whom resection and reconstruction of these vessels were required. In both cases, the gastroepiploic artery was anastomosed to the gastroduodenal artery, and the gastroepiploic vein to the splenic vein. Previous reports have likewise described using the middle colic artery to reconstruct the gastroepiploic artery.^8,12^ Although arterial reconstruction is essential to avert conduit ischemia, the necessity of reconstructing the gastroepiploic vein remains controversial; several reports have documented its resection without subsequent venous reconstruction.^13,14^ It may be feasible to judge the need for venous reconstruction by evaluating the conduit for venous congestion.

A prior report documented a successful case of laparoscopic distal pancreatectomy performed after esophagectomy.^15^ In the present study, all distal pancreatectomies were safely performed using a minimally invasive approach without any complications. In our experience, a minimally invasive approach is feasible for distal pancreatectomy, even after esophagectomy using a gastric conduit. In patients with gastric conduit, the conduit is pulled up straight toward the thoracic cavity or anterior thoracic wall. The stomach should be retracted cranially and anteriorly to dissect the cranial edge of the pancreas in patients without a history of gastrectomy or esophagectomy,^16^ whereas it is not necessary to retract the gastric conduit to do so in patients after esophagectomy. However, it requires adhesiolysis around the cranial edge of the pancreas, because lymphadenectomy around the common hepatic artery and splenic artery is generally performed during esophagectomy for esophageal cancer.

In the present study, the long-term prognosis of patients undergoing PDAC resection after esophagectomy for esophageal cancer was poor. One likely explanation is the low proportion of patients who received adjuvant chemotherapy; in our series, no patient who underwent pancreaticoduodenectomy received post-operative chemotherapy. A key reason for this shortfall is the profound malnutrition and sarcopenia that frequently follow esophagectomy,^17^ which can render patients intolerant of adjuvant chemotherapy after pancreatectomy. For patients with PDAC in the pancreatic head after esophagectomy, treatment strategies other than surgery followed by adjuvant chemotherapy—such as total neoadjuvant therapy followed by resection^18^—may yield superior outcomes. In patients with a prior esophagectomy, the indication for surgery should be assessed cautiously, with particular consideration of the feasibility of perioperative chemotherapy. Another factor suggested by our findings is the intrinsically high malignant potential of PDAC in this cohort. Although more than half of the tumors were detected within 5 years of post-operative surveillance after esophagectomy for esophageal cancer, pathological lymph node metastasis was present in seven patients (77.8%). These observations indicate that the biological behavior of PDAC may differ between patients with and without a prior history of esophageal cancer.

PDAC and esophageal cancer share several environmental and genetic risk factors. Notably, alcohol consumption and cigarette smoking are major risk factors for both malignancies.^19,20^ Aldehyde dehydrogenase 2 dysfunction is reported to be associated with tumorigenesis in the both cancers.^21,22^ Similarly, breast cancer gene 2 mutation is associated with both cancers.^23,24^ Genetic associations between PDAC and esophageal cancer warrant further investigation in future studies.

This study has several limitations. First, it was a single-center, retrospective analysis that included only nine patients. Although the current standard of care for PDAC consists of neoadjuvant therapy followed by pancreatectomy and subsequent adjuvant therapy, only a small proportion of patients in our cohort received either neoadjuvant or adjuvant chemotherapy. Administering adjuvant chemotherapy was particularly challenging for those who underwent pancreaticoduodenectomy after esophagectomy. A more comprehensive treatment approach—such as total neoadjuvant therapy—may improve long-term outcomes in this setting.

In conclusion, pancreatectomy for PDAC can be performed safely in patients with a prior esophagectomy for esophageal cancer; however, long-term survival remains poor, especially after pancreaticoduodenectomy. Therefore, the indication for pancreatectomy in patients with pancreatic-head PDAC after esophagectomy should be weighed carefully, with specific attention to the feasibility of delivering perioperative chemotherapy.

## Supplementary Information

Below is the link to the electronic supplementary material.

Supplementary file1 (DOCX 11 kb)
