# Supplementary material for: Short- and Long-Term Outcomes after Pancreatectomy for Pancreatic Cancer in Patients with Prior Esophagectomy for Esophageal Cancer
Source: Ann Surg Oncol. 2025 Oct 14;33(2):918–24. doi: 10.1245/s10434-025-18491-3 (PMC12765746; doi:10.1245/s10434-025-18491-3)

**Supplementary Information:**

Online Resource 1 (Figure S1). Kaplan–Meier curves of overall survival after pancreaticoduodenectomy in post-esophagectomy patients (n = 5) and controls (n = 147). Red line, post-esophagectomy patients; blue line, controls.


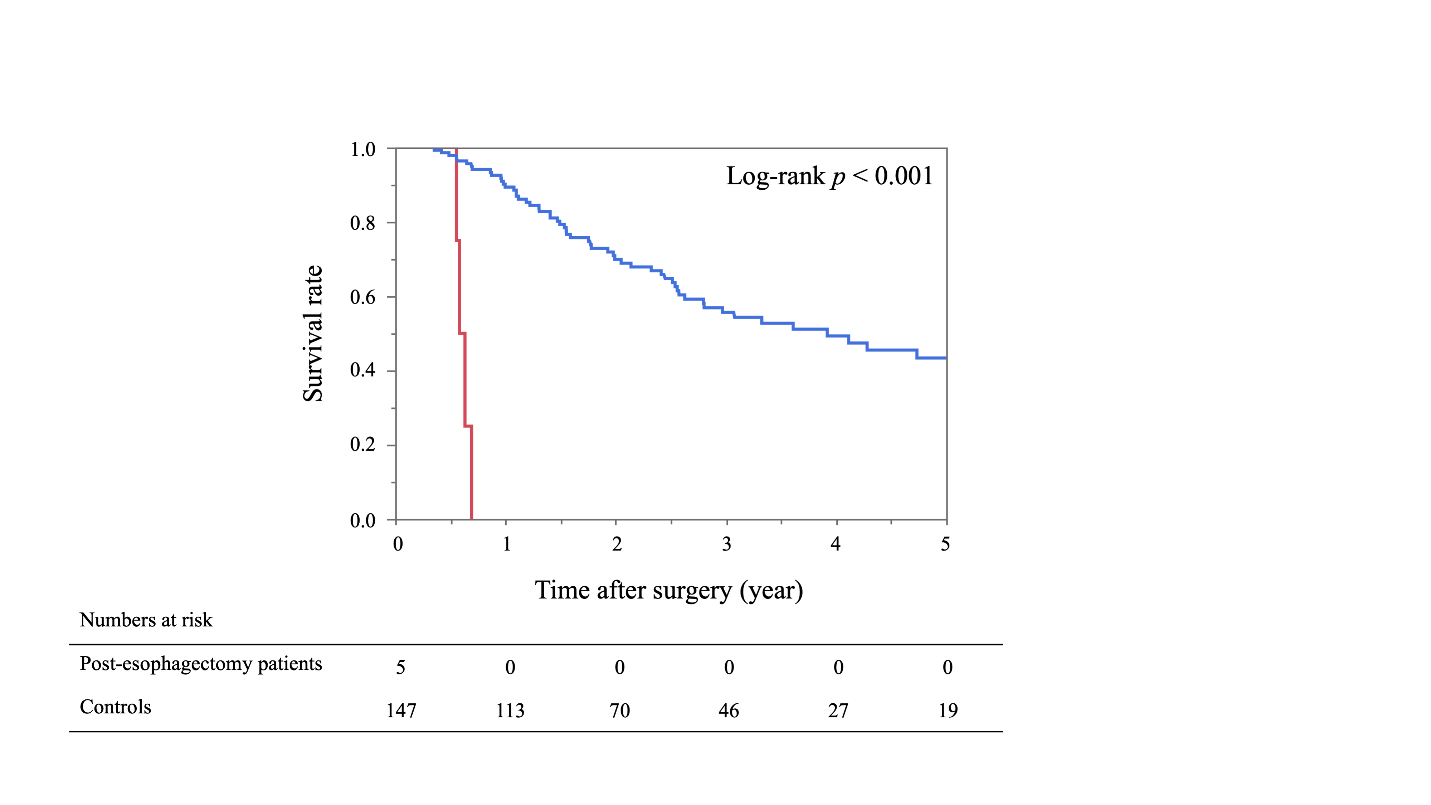


Online Resource 2 (Figure S2). Kaplan–Meier curves of overall survival after distal pancreatectomy in post-esophagectomy patients (n = 4) and controls (n = 102). Red line, post-esophagectomy patients; blue line, controls.


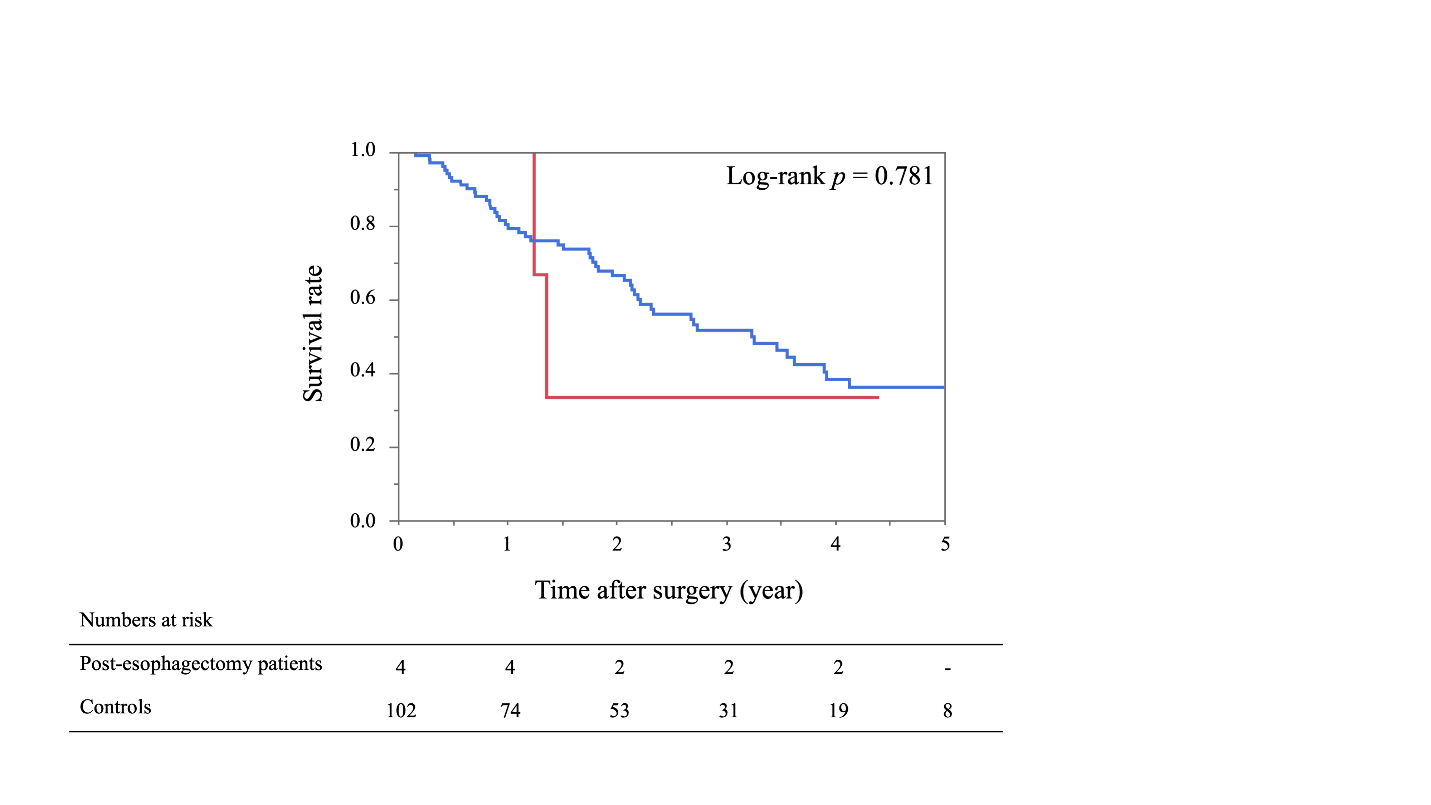

Supplement: Supplementary file 1 — Supplementary file1 (DOCX 11 kb) [file 10434_2025_18491_MOESM1_ESM.docx]
